# Asiaticoside–nitric oxide synergistically accelerate diabetic wound healing by regulating key metabolites and SRC/STAT3 signaling

**DOI:** 10.1093/burnst/tkaf009

**Published:** 2025-03-10

**Authors:** Xingrui Mu, Jitao Chen, Huan Zhu, Junyu Deng, Xingqian Wu, Wenjie He, Penghui Ye, Rifang Gu, Youzhi Wu, Felicity Han, Xuqiang Nie

**Affiliations:** Key Laboratory of Basic Pharmacology of Ministry of Education and Joint International Research Laboratory of Ethnomedicine of Ministry of Education, Zunyi Medical University, No. 6 West Xuefu Road, Xinpu New District, Zunyi 563006, China; College of Pharmacy, Zunyi Medical University, No. 6 West Xuefu Road, Xinpu New District, Zunyi 563006, China; Key Laboratory of Basic Pharmacology of Ministry of Education and Joint International Research Laboratory of Ethnomedicine of Ministry of Education, Zunyi Medical University, No. 6 West Xuefu Road, Xinpu New District, Zunyi 563006, China; College of Pharmacy, Zunyi Medical University, No. 6 West Xuefu Road, Xinpu New District, Zunyi 563006, China; Key Laboratory of Basic Pharmacology of Ministry of Education and Joint International Research Laboratory of Ethnomedicine of Ministry of Education, Zunyi Medical University, No. 6 West Xuefu Road, Xinpu New District, Zunyi 563006, China; College of Pharmacy, Zunyi Medical University, No. 6 West Xuefu Road, Xinpu New District, Zunyi 563006, China; Key Laboratory of Basic Pharmacology of Ministry of Education and Joint International Research Laboratory of Ethnomedicine of Ministry of Education, Zunyi Medical University, No. 6 West Xuefu Road, Xinpu New District, Zunyi 563006, China; College of Pharmacy, Zunyi Medical University, No. 6 West Xuefu Road, Xinpu New District, Zunyi 563006, China; Key Laboratory of Basic Pharmacology of Ministry of Education and Joint International Research Laboratory of Ethnomedicine of Ministry of Education, Zunyi Medical University, No. 6 West Xuefu Road, Xinpu New District, Zunyi 563006, China; College of Pharmacy, Zunyi Medical University, No. 6 West Xuefu Road, Xinpu New District, Zunyi 563006, China; Key Laboratory of Basic Pharmacology of Ministry of Education and Joint International Research Laboratory of Ethnomedicine of Ministry of Education, Zunyi Medical University, No. 6 West Xuefu Road, Xinpu New District, Zunyi 563006, China; College of Pharmacy, Zunyi Medical University, No. 6 West Xuefu Road, Xinpu New District, Zunyi 563006, China; Key Laboratory of Basic Pharmacology of Ministry of Education and Joint International Research Laboratory of Ethnomedicine of Ministry of Education, Zunyi Medical University, No. 6 West Xuefu Road, Xinpu New District, Zunyi 563006, China; College of Pharmacy, Zunyi Medical University, No. 6 West Xuefu Road, Xinpu New District, Zunyi 563006, China; University Medical Office, Zunyi Medical University, No. 6 West Xuefu Road, Xinpu New District, Zunyi 563006, China; Australian Institute for Bioengineering and Nanotechnology (AIBN), The University of Queensland, St. Lucia, Brisbane, QLD 4072, Australia; Australian Institute for Bioengineering and Nanotechnology (AIBN), The University of Queensland, St. Lucia, Brisbane, QLD 4072, Australia; Key Laboratory of Basic Pharmacology of Ministry of Education and Joint International Research Laboratory of Ethnomedicine of Ministry of Education, Zunyi Medical University, No. 6 West Xuefu Road, Xinpu New District, Zunyi 563006, China; College of Pharmacy, Zunyi Medical University, No. 6 West Xuefu Road, Xinpu New District, Zunyi 563006, China; Australian Institute for Bioengineering and Nanotechnology (AIBN), The University of Queensland, St. Lucia, Brisbane, QLD 4072, Australia

**Keywords:** Asiaticoside, Nitric oxide, Metabolomics, Network pharmacology, Diabetic wounds, SRC/STAT3 signaling

## Abstract

**Background:**

Diabetic wounds pose significant clinical challenges due to impaired healing processes, often resulting in chronic, nonhealing ulcers. Asiaticoside (AC), a natural triterpene derivative from *Centella asiatica*, has demonstrated notable anti-inflammatory and wound-healing properties. However, the synergistic effects of nitric oxide (NO)—a recognized promoter of wound healing—combined with AC in treating diabetic wounds remain inadequately explored.

**Methods:**

Ultraperformance liquid chromatography–tandem mass spectrometry (UPLC-MS/MS) was utilized to identify differential metabolites and dysregulated metabolic pathways associated with diabetic wounds. Molecular docking analyses were conducted to confirm the binding affinity of AC to key therapeutic targets. The effects of asiaticoside–nitric oxide hydrogel (ACNO) on gene and protein expression were evaluated using reverse transcription-quantitative polymerase chain reaction (RT-qPCR) and western blotting. *In vitro* experiments using sarcoma (SRC) agonists and inhibitors were performed to investigate the impact of ACNO therapy on the expression of SRC, STAT3, and other proteins in HaCaT cells.

**Results:**

Metabolomic profiling revealed that diabetic wounds in mice exhibited marked metabolic dysregulation, which was attenuated by ACNO treatment. Key metabolites modulated by ACNO included mandelic acid, lactic acid, and 3-hydroxyisovaleric acid. The primary metabolic pathways involved were methyl histidine metabolism and the malate–aspartate shuttle. Immunofluorescence staining confirmed that ACNO therapy enhanced angiogenesis, promoted cellular proliferation, and facilitated diabetic wound closure. RT-qPCR data demonstrated that ACNO regulated the transcription of critical genes (*SRC*, *STAT3*, *EGFR*, and *VEGFA*). Notably, ACNO attenuated SRC/STAT3 pathway activation while concurrently upregulating EGFR and VEGFA expression.

**Conclusions:**

These findings emphasize the therapeutic potential of ACNO hydrogel in diabetic wound healing through the modulation of metabolic pathways and the SRC/STAT3 signaling axis. By correlating altered metabolites with molecular targets, this study elucidates the pharmacodynamic foundation for ACNO’s preclinical application and provides valuable insights into the development of targeted therapies for diabetic wound management.

## Graphical Abstract

**Graphical Abstract:**
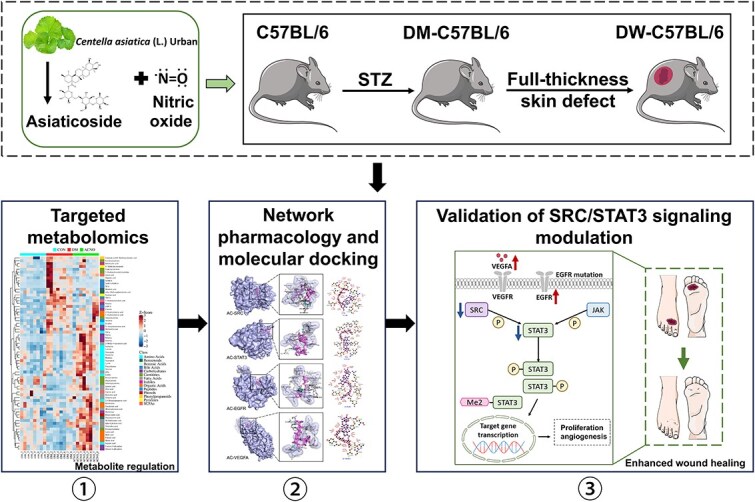

Highlights

- Integrated network pharmacology and metabolomics identify skin biomarkers for ACNO in diabetic wound therapy.
- ACNO hydrogel fosters angiogenesis and re-epithelialization, accelerating diabetic wound healing.
- The underlying mechanisms involve the regulation of key metabolites and SRC/STAT3 signaling.

## Background

Diabetes is a metabolic disorder characterized by various causes, and there is a global increase in the prevalence of diabetes mellitus (DM). Among the >100 complications associated with diabetes, diabetic wounds (DWs) affect ~20% of patients with diabetes and are well-known complications [1]. DWs are distinguished by impaired healing responses, persistent/chronic inflammation, and reduced epithelial formation kinetics among diabetic patients. The wound-healing process is a complex, multistage progression that includes hemostasis, inflammation, proliferation, and remodeling [2]. Compared to normal wounds, DWs have a slower healing rate due to the longer duration of the disease and the complex mechanisms involved, significantly impacting the morbidity, mortality, and quality of life of patients [3].

Wound healing represents a complex and dynamic process that is finely orchestrated by the intricate interaction of numerous cell types across distinct yet overlapping phases. This elaborate cascade is typically classified into four stages: hemostasis, inflammation, proliferation, and remodeling [4]. Once an injury occurs, the hemostatic phase commences immediately, featuring platelet aggregation and the subsequent formation of a fibrin clot. This procedure not only stops blood loss but also releases a multitude of growth factors and cytokines, effectively laying the foundation for the subsequent phases of healing [5]. The subsequent inflammatory phase witnesses the recruitment of neutrophils and macrophages to the wound site. These cells play a crucial role in clearing debris and pathogens while concurrently secreting factors that further promote tissue repair [6].

The proliferative phase then follows, marked by a significant increase in fibroblast, endothelial cell, and keratinocyte proliferation. This cellular activity underpins the formation of granulation tissue, angiogenesis for restoring vascular supply, and re-epithelialization for restoring the skin barrier [7]. Finally, the remodeling phase, often lasting from months to years, witnesses the reorganization and maturation of the newly formed extracellular matrix. This phase is characterized by collagen cross-linking and tissue contraction, ultimately resulting in scar formation [8]. However, in the context of chronic diseases such as diabetes, this delicately tuned process is frequently disrupted, leading to delayed wound healing or the development of chronic wounds [9].

Asiaticoside (AC) is a natural compound extract from *Centella asiatica* (L.) Urban*,* a traditional Chinese medicine used for the treatment of various skin conditions, including skin ulcers and intractable wounds [10]. AC has been shown to promote local leukocytosis, connective tissue proliferation, hair and tail growth, and protect endothelial cells from oxidative damage [11,12]. In our previous research phase, our group successfully combined AC with nitric oxide (NO) to produce a novel hydrogel called asiaticoside–nitric oxide hydrogel (ACNO). ACNO possesses several advantages for wound healing, including inhibiting the growth of bacteria on the wound surface, alleviating inflammatory response in the wounds, upregulating the expression of CD34, endothelial NO synthase (eNOS), inducible NO synthase (iNOS), and vascular endothelial growth factor (VEGF), as well as promoting wound healing in diabetes by regulating the Wnt/β-catenin signaling pathway. These findings indicate that ACNO holds promise as an effective wound treatment option [13].

NO is a fundamental signaling molecule that exhibits pivotal functions in various physiological and pathological conditions. Endogenous NO is produced by the catalytic substrate *L*-arginine of nitric oxide synthase (NOS). At the same time, exogenous NO molecules released by NO donor drugs are primarily used for the clinical treatment of angina pectoris [14]. In addition, NO has been shown to inhibit inflammatory reactions and vasoconstriction and is widely used in the treatment of various skin conditions, including psoriasis, lupus erythematosus, and inflammatory skin diseases [15]. Treatment with a local NO donor did not affect normal epidermal hyperplasia. However, in a damaged epidermal barrier state, NO donors promoted epidermal hyperplasia, suggesting that skin condition influences the effect of local NO donors on epidermal hyperplasia [16].

Metabolomics is an approach for quantitatively analyzing all metabolites in organisms to identify the relative relationship between metabolites and physiological and pathological changes [17]. Targeted metabolome has the characteristics of strong specificity, high detection sensitivity, and accurate quantification [18]. Specific metabolites were detected and analyzed pertinently and specifically with reference to standard substances [19]. Recently, metabolomics has gained increasing attention in the analysis of unconventional biological matrices, such as skin. Low-molecular-weight compounds existing on or in the skin are derived from sweat, sebum, and protein-degrading tissue fluid in the skin’s outer layer, accounting for 45% of human skin [20, 21].

**Figure 1:**
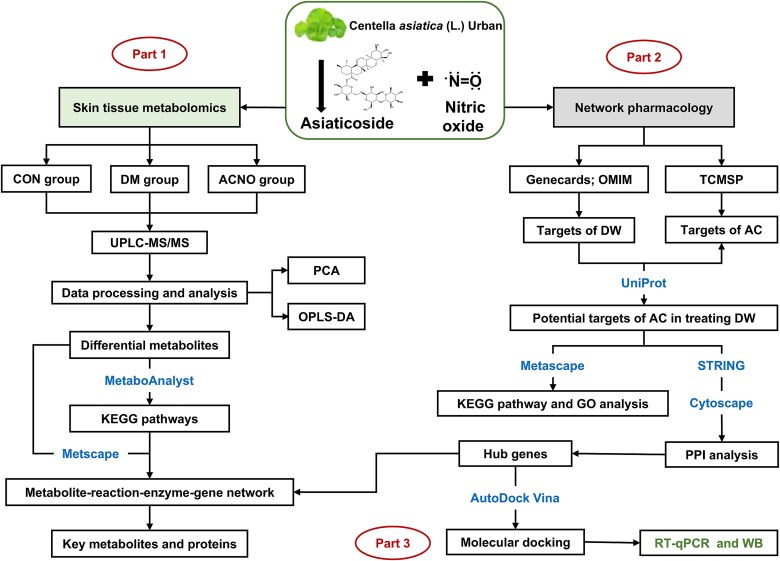
The flowchart of the integrated methods. The mechanisms of ACNO in treating DW were explored with metabolomics of skin tissues (Part 1). Network pharmacology was performed to extract the hub gene (Part 2). Through Parts 1 and 2, important targets and metabolites were identified and connected. Molecular docking and RT-qPCR were conducted to further verify these core targets (Part 3). *ACNO* asiaticoside–nitric oxide hydrogel, *AC* asiaticoside, *DM* diabetes mellitus, *DW* diabetic wound, *UPLC-MS/MS* ultraperformance liquid chromatography–tandem mass spectrometry, *PCA* principal component analysis, *OPLS-DA* OPLS-discriminant analysis, *KEGG* Kyoto Encyclopedia of Genes and Genomes, *PPI* protein–protein interaction

Network pharmacology employs computer simulation algorithms and network analysis to unveil the intricate signal network interplay amid drug–target disease [22, 23]. In this study, the combination of network pharmacology and targeted metabolomics, based on ultraperformance liquid chromatography–tandem mass spectrometry (UPLC-MS/M, was utilized to explore skin biomarkers for the treatment of DWs in patients with AC. Figure 1 depicts the research flowchart.

## Methods

### Preparation of ACNO hydrogel

The preparation of ACNO was synthesized as described in our earlier study [13] and summarized as follows. Glue A contains asiaticoside (1%), ascorbic acid (8%), hydroxyethyl cellulose (1%), glycerol (5%), azone (1%), and ethyl-*p*-hydroxybenzoate (0.2%). PBS is added for dilution to prepare 100 ml of Glue A, followed by mixing with a magnetic stirrer for 1.5 h. Similarly, Glue B comprises sodium nitrite (2%), hydroxyethyl cellulose (1%), glycerol (5%), azone (1%), and ethyl-*p*-hydroxybenzoate (0.2%). PBS is added for dilution to prepare 100 ml of Glue B and mixed with a magnetic stirrer for 1.5 h.

### Chemicals and reagents

Asiaticoside was purchased from Yuanye (#B20586, Shanxi, China). Hydroxyethyl cellulose (#H104788), azone (#A104374), and sodium nitrite (#S111979) were obtained from Shanghai Aladdin Biochemical Technology Co., Ltd (Shanghai, China). Glycerol (#G810575) and ethyl nipagin (#E808808) were purchased from Shanghai McLean Biochemical Technology Co., Ltd (Shanghai, China).

The standards utilized in this study were procured from Sigma-Aldrich (St. Louis, MO, USA), Steraloids Inc. (Newport, RI, USA), and TRC Chemicals (Toronto, ON, Canada). Each standard underwent meticulous weighing before being prepared in solutions of aqueous, methanolic, sodium hydroxide, or hydrochloric acid nature, resulting in individual stock solutions possessing a concentration of 5.0 mg/ml. By judiciously combining appropriate volumes of the aforementioned stock solutions, the requisite stock calibration solutions were successfully synthesized.

Optima™ LC–MS-grade formic acid (cat. No. A117) was acquired from Sigma-Aldrich (St. Louis, MO, USA). In contrast, Optima™ LC-MS-grade methanol (cat. No. A456), acetonitrile (cat. No. A955), and isopropanol (cat. No. A461) were obtained from Thermo-Fisher Scientific (FairLawn, NJ, USA). The ultrapure water, on the other hand, was generated utilizing a Mill-Q reference system that was equipped with an LC-MS Pak filter, sourced from Millipore (Billerica, MA, USA).

### Establishment of diabetic wound model in mice

A total of 24 specific pathogen-free (SPF) C57BL/6 J mice, comprising equal numbers of males and females, with an average weight of 25 ± 3 g, was procured from the Experimental Animal Center of Zunyi Medical University. Following a 1-week period of adaptive feeding, the mice were randomly assigned to one of three groups: a control group (CON, *n* = 8), a model group (DM, *n* = 8), and an AC–NO group (ACNO, *n* = 8). Subsequently, each C57BL/6 J mouse received an intraperitoneal injection of 75 mg/kg streptozotocin (STZ, #V900890, Sigma-Aldrich) in a citric acid buffer (0.1 mol/L, pH 4.5) over the course of 3 days. The streptozotocin was prepared by dissolving it in a citric acid–sodium citrate buffer at a concentration of 1%. This preparation process was conducted in an ice bath under dark conditions, and all injections were completed within 30 min. Following a subsequent interval of 72 h, blood samples were collected from the tail vein to assess fasting glucose levels using a glucose meter (One Touch Ultra; Johnson & Johnson, New Brunswick, NJ, USA). The DM model was deemed successfully established upon reaching a blood glucose concentration of ≥16.7 mmol/L [24, 25]. Subsequently, typical indications of polydipsia, polyphagia, polyuria, and weight loss manifested within the ensuing 2 weeks.

A DW model was created on mice by anesthetizing the animals with 1.25% 2,2,2- tribromoethanol (i.p., 24 ml/kg), shaving and sterilizing the dorsum. Next, the back was cleaned with 75% medical alcohol before a 1.0 cm diameter circular full-thickness skin defect was made on each side of the mouse back using surgical scissors, extending to the subcutaneous level to create a skin wound model. The wounds were left to heal naturally for one day, with the second day recorded as the first day of the experiment. This study was approved by the Ethics Committee of Zunyi Medical University (Approval no. ZMU21-2107-037).

### Animals and treatment

The mice received different treatments as follows: (i) control group and model group: each wound was coated with 0.1 ml of normal saline twice a day. (ii) ACNO group: local administration was performed twice daily, ACNO hydrogel was transferred to the wound surface of mice with a 1 ml syringe, and the dosage of each wound was 0.1 ml (0.05 ml for glue A and glue B, respectively). All groups were treated continuously for 14 days and then sampled.

### Cell culture and experimental reagents

Human immortalized keratinocytes (HaCaT, obtained from the National Collection of Authenticated Cell Cultures, Kunming, China, No.: KCB, 200442 YJ) were cultured in a Dulbecco's Modified Eagle Medium (DMEM) (Gibco, China) medium supplemented with 10% fetal bovine serum (FBS), 100 U/ml penicillin and 100 μg/ml streptomycin. All cells were cultured in a 5% CO_2_ humidified atmosphere at 37°C. Cells were then exposed to 40 mM glucose to establish a high-glucose model where the ACNO treatment group was given AC (240 μM, Yuanye, China) and SNP (2.5 μM, Macklin, China). The sarcoma (SRC) agonist, tolimidone (10 μM, MedChemExpress, USA), or the SRC inhibitor, PP2 (10 μM, Beyotime, China), was added to cells cultured.

### Hematoxylin–eosin and Masson’s staining

After 7 and 14 days of continuous administration, the skin tissue from the mouse wounds was fixed in a 4% paraformaldehyde solution. Paraffin tissue sections were prepared and stained with hematoxylin–eosin (H&E; G1003, Servicebio, Wuhan) and Masson (G1006, Servicebio, Wuhan). The morphological changes of the wounds were observed using an optical microscope (Olympus, Olympus DX51).

### Immunofluorescence staining

Tissue samples were dehydrated and embedded in paraffin. Then, antigen repair was carried out, a circle was drawn around the skin tissue, and it was sealed with serum. First, the target proteins (CD31, α-SMA, and Ki67, all from Servicebio, Wuhan) were detected by specific antibodies. DAPI dye solution (G1012, Servicebio, Wuhan) was added to stain the nucleus again, then, the autofluorescence quencher (G1221, Servicebio, Wuhan) was added, and finally, the film was sealed for observation.

### Sample collection and preparation

The tissue samples were washed with PBS solution or normal saline three to five times, and the excess liquid was removed with filter paper. The samples were arranged within an Eppendorf Safe-Lock microcentrifuge tube, rapidly cooled in liquid nitrogen for a duration of 15 min and then conserved at a temperature of −80°C.

Each tissue sample, weighing ~10 mg, was carefully procured and subsequently placed into an Eppendorf Safe-Lock microcentrifuge tube. The samples were then combined with 10 prechilled zirconium oxide beads and supplemented with an aliquot of 20 μl deionized water. Following this preparation, the sample underwent homogenization for a duration of 3 min. After the homogenization process, the sample was treated with 120 μl of methanol containing an internal standard to facilitate metabolite extraction. The sample was subjected to homogenization for an additional 3 min, after which it was centrifuged at 18 000 g for 20 min. The resulting supernatant was then introduced to a 96-well plate and treated with an Eppendorf epMotion® Workstation (Eppendorf Inc., Hamburg, Germany). To achieve optimal derivatization, freshly synthesized derivative reagents (20 μl) were incorporated into each well, followed by hermetic sealing of the plate for 60 min at 30°C. Following derivatization, the sample underwent evaporation for 2 h.

Thereafter, a 50% methanol solution (330 μl), maintained at an ice-cold temperature, was added to the plate to restore the sample. The plate was stored in a temperature-controlled environment at−20°C for 20 min, after which it was centrifuged at 4000 g for 30 min at 4°C. The resulting supernatant (135 μl) was subsequently transferred to a new 96-well plate, with each well containing 10 μl of internal standards. Finally, the remaining wells were filled with serial dilutions of derivatized stock standards, and the plate was sealed for LC-MS analysis [26].

### Chromatographic conditions and mass spectrometry conditions

The present project utilized the ACQUITY UPLC-Xevo TQ-S, manufactured by Waters Corp, as the UPLC-MS/MS system. The experimental chromatographic conditions encompassed the utilization of two columns: an ACQUITY UPLC BEH C18 1.7 μM vanguard precolumn (2.1 × 5 mm) and an ACQUITY UPLC BEH C18 1.7 μM analytical column (2.1 × 100 mm). The experimental method was performed as described in previous studies [27, 28] and is briefly described below. The mobile phase was formulated by two solvents, namely, Solvent A, constituted by water containing 0.1% formic acid, and Solvent B, which was a mixture of acetonitrile and IPA (70:30). The injection volume was established at 5 μl while maintaining the column temperature at 40°C. Additionally, the sample manager temperature was meticulously regulated to 10°C, and the flow rate was fixed at 0.4 ml/min. The gradient conditions for elution of the metabolites were as follows: 0–1 min (5% B), 1–11 min (5%–78% B), 11–13.5 min (78%–95% B), 13.5–14 min (95%–100% B), 14–16 min (100% B), 16–16.1 min (100%–5% B), and 16.1–18 min (5% B). To elaborate further, the methodology utilized was mass spectrometry (MS) in conjunction with electrospray ionization (ESI) as the source, wherein both positive and negative ion modes were employed. The application of capillary voltage was set at 1.5 kV for ESI+ and 2.0 kV for ESI−, while the temperature of the source was configured at 150°C and the desolvation temperature at 550°C. Lastly, the desolvation gas flow rate was established at 1000 L/hour [29].

### Data processing and multivariate data analysis

The raw data files acquired from UPLC-MS/MS were subjected to peak integration, calibration, and quantitation of each metabolite employing TMBQ software (v1.0, Metabo-Profile, Shanghai, China). In addition, statistical analysis was carried out using the iMAP platform (v1.0, Metabo-Profile, Shanghai, China) [30, 31]. This article encompasses different methodologies, namely, principal component analysis (PCA), OPLS-discriminant analysis (OPLS-DA), univariate analysis, and pathway analysis. PCA was utilized to detect outliers in mouse skin tissue samples and to differentiate the similarities and differences in metabolic characteristics of tissue samples in each group. For OPLS-DA analysis, the Ropls package in R software was used, and the quality of the OPLS-DA model was assessed using R^2^Y (cum) and Q^2^Y (cum) parameters. The differential metabolites were identified using the intersection of 1D test *P* < .05, |log_2_FC| ≥ 0, and multidimensional statistical variable importance for projection statistics (VIP > 1) as default criteria. Finally, the metabolic pathway was determined according to the differential metabolites using a significance threshold of *P* < .05.

### Methods of network pharmacology

The candidate gene for asiaticoside was obtained from the Traditional Chinese Medicine Systems Pharmacology Database and Analysis Platform (TCMSP) (https://www.tcmspw.com), which is a comprehensive platform for the systematic pharmacology of traditional Chinese medicine. The UniProt (https://www.uniprot.org/) database was used to standardize gene names and eliminate duplicate and nonhuman target genes, obtaining candidate target genes of pharmaceutical ingredients. DW-related genes were obtained from the Gene Cards (https://www.genecards.org/) database, and the Online Mendelian Inheritance in Man (OMIM) (https://www.omim.org/) database was used to eliminate duplicate genes and supplement DW–related genes. A Venn diagram was drawn to identify the potential target for treating DWs by taking the intersection target of the asiaticoside candidate gene and DW candidate gene. The utilization of the Search Tool for the Retrieval of Interacting Genes/Proteins (STRING) database (https://string-db.org/) facilitated the creation of a protein–protein interaction (PPI) network, which was founded upon the potential targets of AC and DWs and deliberated on interactions that possessed the highest level of confidence, specifically at 0.900. The data were imported into the Cytoscape software (v3.9.1) in tab-separated value (TSV) file format, which enabled the visualization of the PPI map. Furthermore, the Metascape database (http://metascape.org/) was employed for Gene Ontology (GO) function enrichment and Kyoto Encyclopedia of Genes and Genomes (KEGG) pathway analyses. Statistical significance was considered when *P* < .01 and the enrichment score > 1.5. The top 10 GO items were visualized as a histogram, and the top 20 KEGG items were represented as a bubble chart. Drawing and other modules are written in R/Python and other languages or drawn by https://www.bioinformatics.com.cn (an online platform for data analysis and visualization).

Differential metabolites from metabolomics analysis and target genes from network pharmacology were introduced into Cytoscape software (v3.9.1). The network diagram of “metabolite-reaction-enzyme-gene” was constructed using the plug-in MetScape (v3.1.3).

### Molecular docking

The structural file in 2D of asiaticoside was obtained from the PubChem database, which is a comprehensive resource for chemical information (https://pubchem.ncbi.nlm.nih.gov/). The structure of AC was optimized by Chem3D software (v20.0). The 3D structure of the target protein was acquired from the Research Collaboratory for Structural Bioinformatics Protein Data Bank (RCSB PDB) (https://www.pdbus.org/). PyMOL software (v2.2.0) was utilized to remove water molecules and residues from the protein structure.

To predict the active pockets of SRC, STAT3, EGFR, and VEGFA, the DeepSite (https://www.playmolecule.com/deepsite/) and the DoGSiteScorer server (https://proteins.plus/) were utilized, and the coordinates of the active sites were obtained. Finally, the conduction of molecular docking was executed through the utilization of AutoDock Vina software (v1.1.2). The consequences were subsequently exhibited through PyMOL software (v2.2.0) and LigPlot+ (v2.2.8).

### RT-qPCR

Total RNA was extracted using Trizol (Beyotime, Shanghai, China, Cat# R0016) from the skins of C57BL/6 J mice in CON, DM, and ACNO groups. Measure the concentration of RNA and D(λ)260/D(λ)280 is 1.8 ~ 2.0. RNA was transcribed in a reverse manner into complementary DNA through the utilization of the PrimeScript™ RT reagent Kit (Perfect Real Time) (Takara, Japan, Cat^#^ RR037A). The real-time fluorescence quantitative PCR system, Bio-Rad CFX-96 (Bio-Rad, USA) was employed for RT-qPCR, utilizing the TB Green® *Premix Ex Taq*™ II (Tli RNaseH Plus) kit (Takara, Japan, Cat^#^ RR820A). The relative expression of the genes was determined by implementing the 2 ^(−△△CT)^ technique, with β-actin as an internal reference. The primers were synthesized by Sangon Biotech (Shanghai, China). The gene primer sequences utilized in this study can be retrieved from Table 1.

**Table 1:** Sequences of RT-qPCR primers

**Gene**	**Forward primer (5′–3′)**	**Reverse primer (5′–3′)**	**Expected amplicon size (base pair)**
MM-*SRC*	GTCACCGCCTCACTACCGTATG	CCACACCTCTCCGAAGCAACC	134
MM-*STAT3*	CGATGCCTGTGGGAAGAGTCTC	ATCTGCTGCTTCTCTGTCACTACG	110
MM-*EGFR*	CCTGATTGGTGCTGTGCGATTC	TGGCAGTTCTCCTCTCCTCCTC	195
MM-*VEGFA*	CCACGACAGAAGGAGAGCAGAAG	GGTCTCAATCGGACGGCAGTAG	86

*MM* Mus musculus, *SRC* sarcoma, *STAT3* signal transducer and activator of transcription 3, *EGFR* epidermal growth factor receptor, *VEGFA* vascular endothelial growth factor A

### Western blotting

The total protein was extracted using RIPA lysis buffer (Solarbio, Beijing, China). The cell lysates were sonicated, and the protein concentration was measured using a BCA Protein Analysis kit (Solarbio, Beijing, China) and an enzyme-labeled instrument (Thermo Scientific, USA). Protein samples (25 μg) were separated using a 10% sodium dodecyl sulfate-polyacrylamide gel electrophoresis (SDS-PAGE) Gel Fast Preparation Kit (Epizyme, Shanghai, China) and transferred onto polyvinylidene difluoride membranes (Millipore, MA, USA) by electrotransfer. After blocking, the membranes were incubated overnight at 4°C with antibodies against SRC (1:1000; #R25792, Zenbio), STAT3 (1:1000; #R22785, Zenbio), EGFR (1:1000; #AF5153, Beyotime), VEGFA (1:1000; #A12303, Abclonal), and β-actin (1:10000; #EM21002, Huabio) antibodies. All antibodies were procured from ABclonal Technology Co., Ltd (Wuhan, China), Beyotime Biotechnology Co., Ltd (Shanghai, China), and HuaAn Biotechnology Co., Ltd (Zhejiang, China). Protein bands were detected using a ChemiDoc™ XRS+ system (Bio-Rad, CA, USA) and developed using an Omni-ECL™Pico Light Chemiluminescence Kit (Epizyme, Shanghai, China). The bands were quantified using Image Lab™ Software (Bio-Rad, CA, USA). The experiments were repeated at least three times to ensure reproducibility.

### Statistical analysis

Statistical analyses were performed using GraphPad Prism software (version 9). The Shapiro–Wilk normality test was used to assess the distribution of the data, and Leven’s test was used to assess intra- and intergroup variability. Paired Student’s *t*-test, one-way paired analysis of variance (ANOVA) corrected by Tukey’s multiple comparison test, and two-way paired ANOVA corrected by Tukey’s multiple comparison test were applied when normality and equal variance were achieved between groups. The data that do not conform to the normal distribution are described by median (Q1, Q3), and the nonparametric test method is used in statistical analysis. *P*-value < .05 was considered to be statistically significant.

## Results

### The diabetic model was successfully established and ACNO can reduce blood glucose in mice

**Figure 2:**
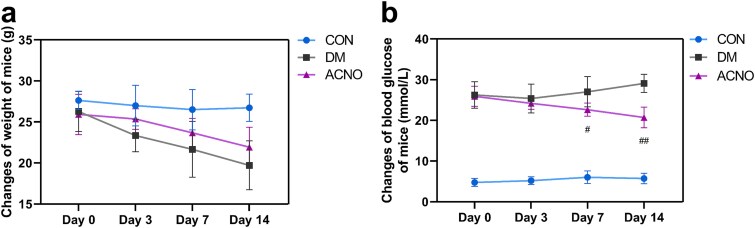
Changes of body weight and blood glucose in mice. (a) Weight changes of mice in different groups at different time periods. (b) Changes in blood glucose in mice of each group at different time periods. ^#^*p*< .05, ^##^*p* < .01, ^###^*p*< .001 *vs* DM. Data are presented as mean ± SD, *n* = 6. *ACNO* asiaticoside–nitric oxide hydrogel, *AC* asiaticoside, *CON* control, *DM* diabetes mellitus

Prior to the establishment of the type 1 diabetes model, the diet and urination patterns of the mice were normal. Following the creation of the diabetic model, there was a marked increase in the mice’s food intake, water consumption, and urine volume, alongside a noticeable decrease in body weight, which exhibited the typical symptoms of “three more and one less.” Body weight monitoring revealed a significant decrease on the 7th and 14th days, as illustrated in Figure 2. Postmodeling, the blood glucose levels of the mice progressively increased, remaining at or above 16.7 mmol/L. These findings indicate that the type 1 diabetes model in mice was successfully established. On the 7th and 14th days, the blood glucose levels in the ACNO group showed a gradual decrease compared to the DM group, with the difference reaching statistical significance.

### ACNO hydrogel therapy can promote the healing of diabetic wounds in mice

**Figure 3:**
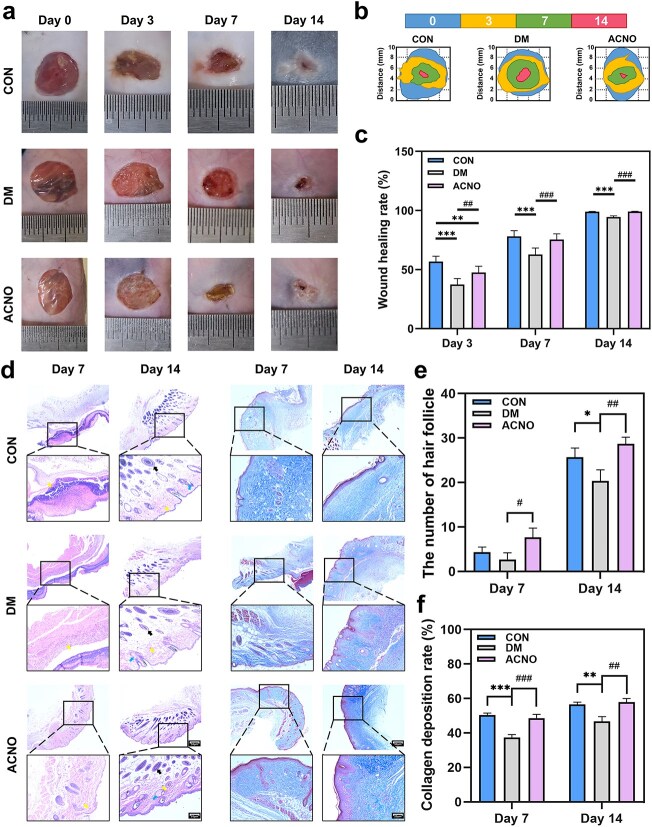
Therapeutic effect and pathological changes of ACNO hydrogel on DWs in mice. (a) The wound-healing process of mice at different times. (b) Redepiction of wound-healing processes. (c) Wound healing rate. ^*^p < .05, ^**^p < .01, ^***^p < .001 *vs* CON; ^#^*p* < .05, ^##^*p* < .01, ^###^*p* < .001 *vs* DM. Data are expressed as mean ± SD, *n* = 8. (d) Hematoxylin–eosin (H&E) and Masson’s staining (H&E × 100, Masson × 100). Black arrow: hair follicles; yellow arrow: fibroblast nuclei; blue arrow: sebaceous glands. Scale bar: 50 μm. (e) The number of hair follicles in different groups at different times. (f) Collagen deposition rate. ^*^p < .05, ^**^p < .01, ^***^p < .001 *vs* CON; ^#^*p* < .05, ^##^*p* < .01, ^###^*p* < .001 *vs* DM. Data are presented as mean ± SD, *n* = 6. *ACNO* asiaticoside–nitric oxide hydrogel, *AC* asiaticoside, *CON* control, *DM* diabetes mellitus

Normalized wound healings are presented in Figure 3**.** The wound healing rate in the DM model group exhibited a relatively slower progression compared to the CON group, particularly on the third and seventh days. Statistical analysis revealed that the wound healing rate of the DM group differed significantly from that of the CON group (*P* < .05), indicating the successful establishment of the DW model. The ACNO group exhibited a higher wound healing rate than the DM group. These findings demonstrate that ACNO can effectively improve the slow healing of DWs. On the seventh day, ACNO hydrogel promoted wound scabs and accelerated wound repair (Figure 3a). On the 14th day, the rate at which wounds healed within the ACNO group was found to be nearly equivalent to that within the CON group and notably more significant than that in the DM group as demonstrated by Figure 3c.

### ACNO hydrogel can promote mouse hair follicle regeneration and increase collagen deposition rate

H&E staining was used to analyze wound tissue in mice on Days 7 and 14 after modeling. As illustrated in Figure 3d, on Day 7, the ACNO group exhibited hair follicles with a continuous and intact epidermal structure alongside significant fibroblast proliferation. In contrast, the DM group displayed an incomplete epidermal structure, a scarcity of hair follicles, and disorganized collagen fibers. By Day 14, all groups demonstrated a complete epidermal structure; however, the number of hair follicles present in the ACNO and CON groups was greater than that observed in the DM group (Figure 3e). Masson’s staining was utilized to stain collagen to a dark blue color, with the intensity of the color serving as an indicator of collagen content and deposition at the wound site. The wound tissue of diabetic mice in the ACNO group demonstrated organized, blue-stained collagen fibers on the wound surface. In contrast, the blue color in the DM group was lighter, with collagen fibers appearing sparse and disordered. Collagen deposition rates on Days 7 and 14 were similar in the ACNO and CON groups and higher than in the DM group (Figure 3f).

### ACNO can up-regulate the expression of CD31, α-SMA, and Ki67 and promote cell proliferation and angiogenesis in diabetic wounds

**Figure 4:**
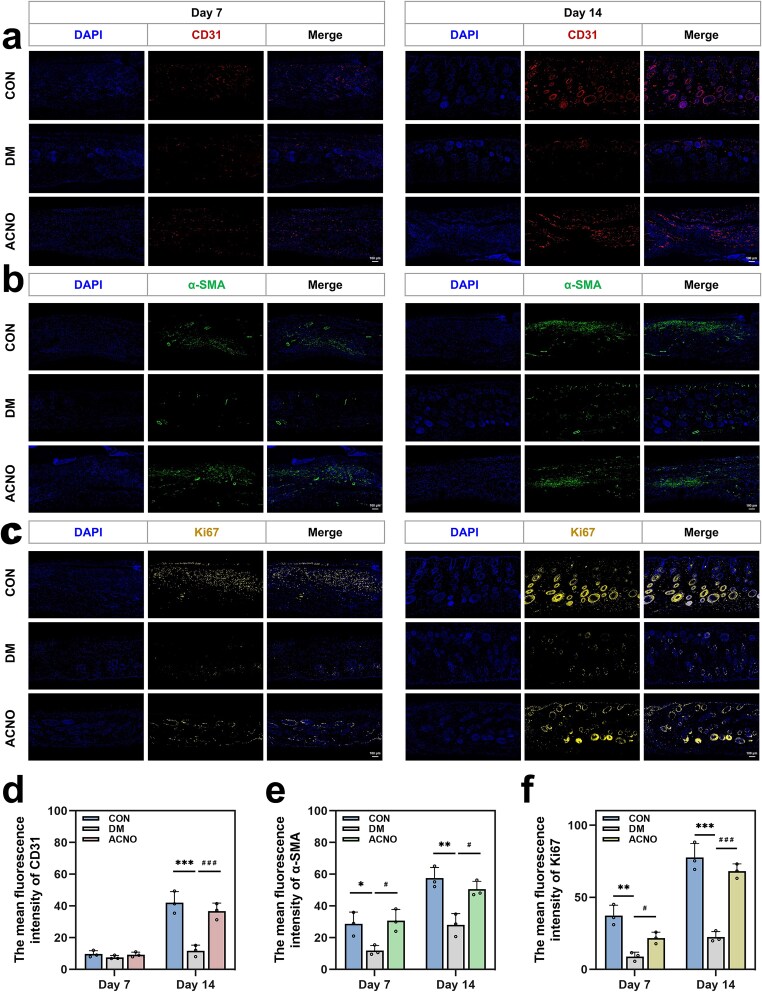
Results and statistics of immunofluorescence staining. (a–c) The results of immunofluorescence staining of CD31 (red), α-SMA (green), and Ki67 (yellow) are shown, respectively. (d–f) The average fluorescence intensity of CD31, α-SMA, and Ki67 were quantitatively analyzed. ^*^p < .05, ^**^p < .01, ^***^p < .001 *vs* CON; ^#^*p* < .05, ^##^*p* < .01, ^###^*p* < .001 *vs* DM. Data are presented as mean ± SD, *n* = 3. *CD31* platelet endothelial cell adhesion molecule-1, *α-SMA* α-smooth muscle actin, *ACNO* asiaticoside–nitric oxide hydrogel, *AC* asiaticoside, *CON* control, *DM* diabetes mellitus

The expression levels of the vascular endothelial-specific marker CD31 and the vascular smooth muscle cell marker α-SMA were assessed to evaluate angiogenesis. Concurrently, the expression level of Ki67 was measured to assess cell proliferation activity. The results of immunofluorescence staining and statistical analysis are presented in Figure 4. On the seventh day, no significant difference in CD31 expression was observed among the three groups. However, on the seventh day, the expression levels of α-SMA and Ki67 in the ACNO group were significantly different compared to the DM group. On the 14th day, the expressions of CD31, α-SMA, and Ki67 in the ACNO group were significantly different when compared to the DM group. Based on the immunofluorescence staining results for CD31, α-SMA, and Ki67, the ACNO group exhibited a higher average fluorescence intensity than the DM group, indicating that ACNO hydrogel can create a favorable microenvironment for angiogenesis and promote DW healing.

### Principal component analysis and nine different metabolites were obtained by targeted metabolomics

**Figure 5:**
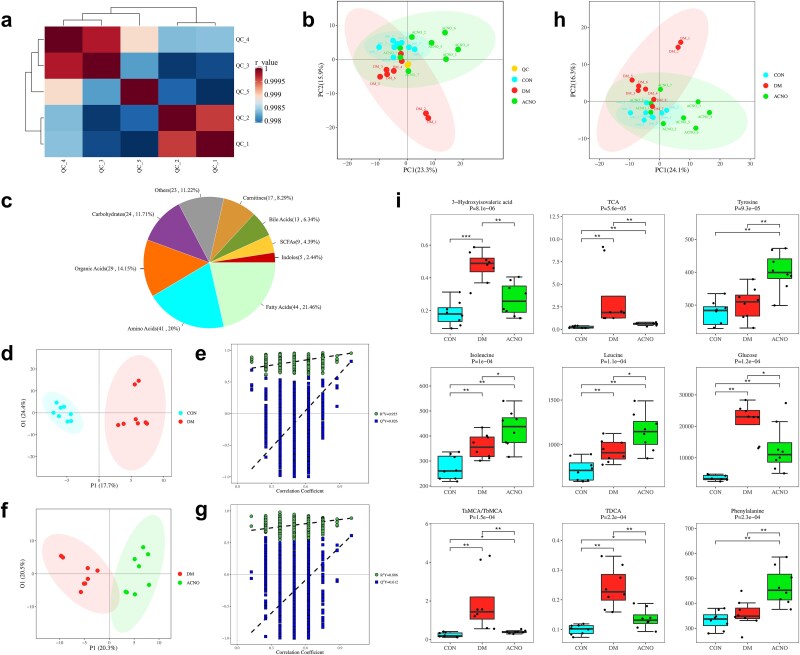
Metabolomics analysis of ACNO hydrogel in mice with DWs. (a) Correlation between quality control (QC) samples. (b) Plot of PCA scores for the CON, DM, and ACNO groups and QC samples. (c) Identification and classification of metabolites. Metabolite classification includes fatty acids, amino acids, organic acids, carbohydrates, carnitines, bile acids, SCFAs, indoles, and others. (d, e) Multivariate data analysis of skin tissue metabolites in mice. OPLS-DA score charts (circles indicate samples with 95% confidence interval) and permutation test charts of CON group and DM group; (f, g) OPLS-DA score chart and permutation test chart of DM group and ACNO group. (h) The plot of PCA scores for the CON, DM, and ACNO groups. (i) Box plot of differential metabolites in the top nine *P*-values for univariate statistical analysis. ^*^p < .05, ^**^p < .01, ^***^p < .001. Data are presented as mean ± SD, *n* = 8. *ACNO* asiaticoside–nitric oxide hydrogel, *AC* asiaticoside, *CON* control, *DM* diabetes mellitus, *PCA* principal component analysis, *OPLS-DA* OPLS-discriminant analysis, *DM* diabetes mellitus

The metabolomics data reported in this paper have been deposited in the OMIX, China National Center for Bioinformation/Beijing Institute of Genomics, Chinese Academy of Sciences (https://ngdc.cncb.ac.cn/omix: accession no. OMIX006890-01). The heat map depicted in Figure 5a displays the correlation coefficient of the QC sample to other QC samples, with each cell’s color indicating the strength of this coefficient. A correlation coefficient near one was observed in this study, indicating that the closer the QC samples were, the greater the test data stability, and hence, a strong quality control effect was achieved in the experimental setup.

Unsupervised PCA was performed on the data from each group, with the CON, DM, and ACNO groups exhibiting significant separation. These results suggest distinct metabolic characteristics among the groups, implying significant changes in endogenous tissue metabolites (Figure 5b). The strong aggregation of QC samples indicates that the overall stability of the analysis system was satisfactory, fulfilling the requirements for metabolomics analysis. A partial overlap between the CON and ACNO groups was observed, indicating that ACNO intervention in the DM model mice tended to bring the metabolic state closer to that of the normal group. The ACNO group was distributed between the CON and DM groups, demonstrating the regulatory effect of ACNO hydrogel on the metabolic disorder caused by DWs.

A total of 205 metabolites were identified and categorized, with their constituent ratio shown in Figure 5c. Fatty acids (44, 21.46%), amino acids (41, 20%), and organic acids (29, 14.15%) were the most prevalent categories among the identified metabolites.

The OPLS-DA technique, which is a supervised discriminant analysis statistical approach, is frequently employed to compare two distinct groups and to detect dissimilarities between them. Within this investigation, partial least squares regression was implemented to establish a connection model that relates the metabolite expression with the sample categories, thereby accomplishing the forecast of sample categories. The cumulative values of parameters R^2^Y (cum) and Q^2^Y (cum) were close to 1.0, indicating that the model was dependable and had satisfactory prediction ability. Generally, R^2^Y and Q^2^Y higher than 0.5 are preferable.

The results of OPLS-DA analysis in the CON and DM groups demonstrated that samples within the same group were closely grouped together, while those from different groups were clearly separated (Figure 5d). The permutation test results for R^2^Y and Q^2^Y were 0.955 and 0.826, respectively (Figure 5e). The OPLS-DA analysis performed on the DM and ACNO groups yielded similar results. Specifically, it was observed that samples from the same group were found to cluster together. In contrast, those from different groups were differentiated (Figure 5f). The permutation tests indicated R^2^Y and Q^2^Y to be 0.886 and 0.612, respectively (Figure 5g). These results indicate that the model has reasonable interpretation and prediction capabilities.

The CON, DM, and ACNO groups were subjected to multidimensional statistics analysis. As shown in Figure 5h, the values of the ACNO group overlap with those of the DM group and CON group, indicating that the DM group can approach the CON group after ACNO intervention. To discern the distinctive metabolites amidst various groups, either a 1D analysis of variance (ANOVA) or Kruskal–Wallis (K-W test) was judiciously chosen, contingent on the normality of the data and homogeneity of variance. The differential metabolite threshold was set to *P* < .05. Box plots of differential metabolites in the top nine *P*-values for univariate statistical analysis are shown in Figure 5i. These nine metabolites are 3-hydroxyisovaleric acid, taurocholate acid (TCA), tyrosine, isoleucine, leucine, glucose, tauro-α-muricholic acid /tauro-β-muricholic acid (TaMCA/TbMCA), tauroursodeoxycholic acid (TDCA), and phenylalanine.

**Table 2:** Different endogenous metabolites in mice skin tissues

**CON/DM**				**DM/ACNO**			
**No.**	**Metabolite**	***P***	**VIP**	**No.**	**Metabolite**	***P***	**VIP**
1	Anserine	.0094	1.5571	1	Lysine	.0031	1.4943
2	Creatine	.0047	1.4421	2	Histidine	.0031	1.4078
3	Lactic acid	.0127	1.5193	3	Glutamine	.0141	1.2587
4	Aspartic acid	.0008	1.9136	4	Anserine	.0499	1.2397
5	Pyrrole-2-carboxylic acid	.0404	1.3863	5	GABA	.0499	1.3981
6	Picolinic acid	.0105	1.6257	6	Lactic acid	.0047	1.5502
7	ortho-Hydroxyphenylacetic acid	.0070	1.4243	7	Tyrosine	.0025	1.4636
8	Hippuric acid	.0003	1.8791	8	Asparagine	.0157	1.0639
9	Malic acid	.0080	1.7215	9	Phenylalanine	.0041	1.4442
10	2-Methy-4-pentenoic acid	.0051	1.4540	10	Aspartic acid	.0018	1.6689
11	Coumaric acid/4-Hydroxycinnamic acid	.0038	1.4778	11	ortho-Hydroxyphenylacetic acid	.0070	1.4340
12	Indoleacetic acid	.0047	1.2171	12	Hippuric acid	.0006	1.9857
13	Fumaric acid	.0048	1.7959	13	Malic acid	.0104	1.5117
14	Indole-3-propionic acid	.0281	1.2393	14	Coumaric acid/4-Hydroxycinnamic acid	.0449	1.5617
15	Galactonic acid	.0172	1.4863	15	Fumaric acid	.0030	1.5622
16	alpha-Aminobutyric acid	.0097	1.2278	16	Ketoleucine	.0379	1.0224
17	*N*-Acetylneuraminic acid	.0064	1.4059	17	Phenylpyruvic acid	.0011	1.6977
18	Acetic acid	.0207	1.3075	18	Melibiose	.0389	1.1864
19	Valine	.0207	1.0711	19	Glucose	.0201	1.6626
20	Glucose	.0002	2.2696	20	Maltotriose	.0070	1.3848
21	Maltose/Lactose	.0240	1.2226	21	3-Hydroxyisovaleric acid	.0010	1.7924
22	3-Hydroxybutyric acid	.0002	1.7967	22	Leucine	.0256	1.1248
23	2-Hydroxybutyric acid	.0047	1.8716	23	Ribulose	.0499	1.0689
24	3-Hydroxyisovaleric acid	.0000	2.1934	24	Xylulose	.0499	1.0005
25	Isoleucine	.0029	1.5735	25	*N*-Acetyl-*D*-glucosamine	.0449	1.0747
26	Leucine	.0041	1.5284	26	Homovanillic acid	.0272	1.3539
27	Xylose	.0281	1.3167	27	*p*-Hydroxyphenylacetic acid	.0281	1.0164
28	Fructose	.0002	2.2000	28	Tryptophan	.0021	1.4600
29	Propionic acid	.0379	1.2866	29	Maleic acid	.0070	1.6065
30	Mandelic acid	.0006	1.6069	30	TwMCA	.0011	1.5767
31	*N*-Methylnicotinamide	.0379	1.0623	31	TaMCA/TbMCA	.0002	1.6736
32	Maleic acid	.0065	1.7523	32	Caproic acid	.0023	1.6471
33	Adipic acid	.0316	1.1148	33	TCA	.0002	1.5958
34	TwMCA	.0011	1.2952	34	TDCA	.0015	1.6038
35	TaMCA/TbMCA	.0002	1.4693	35	Oxoglutaric acid	.0104	1.2286
36	3-Methyladipic acid	.0492	1.2055	36	Octanoic acid	.0379	1.3041
37	TCA	.0002	1.3740	37	5Z-Dodecenoic acid	.0062	1.7782
38	TDCA	.0002	2.1181	38	Myristoleic acid	.0246	1.3306
39	TCDCA	.0047	1.1764	39	9E-tetradecenoic acid	.0148	1.4593
40	wMCA	.0013	1.5347	40	Ricinoleic acid	.0019	1.7331
41	aMCA	.0219	1.6111	41	Palmitoleic acid	.0207	1.4326
42	Oxoglutaric acid	.0281	1.3695	42	alpha-Linolenic acid	.0148	1.6539
43	Ricinoleic acid	.0006	1.3901	43	Linoleic acid	.0085	1.5003
44	Palmitoleic acid	.0379	1.6086	44	Arachidonic acid	.0379	1.0978
45	DPA	.0499	1.2077	45	Oleic acid	.0213	1.4265
46	Oleic acid	.0050	1.7720	46	Propionylcarnitine	.0162	1.7283
47	Propionylcarnitine	.0379	1.5368	47	Decanoylcarnitine	.0054	1.5269
48	Isovalerylcarnitine	.0057	1.6529	48	Tetradecanoylcarnitine	.0499	1.1788
49	3-Hydroxylisovalerylcarnitine	.0002	1.6311	49	Palmitoylcarnitine	.0030	1.2244
50	Adipoylcarnitine	.0014	1.6945	50	Oleylcarnitine	.0047	1.1475
51	Octanoylcarnitine	.0281	1.1844	51	Stearylcarnitine	.0006	1.0867
52	Decanoylcarnitine	.0180	1.5485	52	Fructose 6-phosphate	.0057	1.6378
53	Palmitoylcarnitine	.0148	1.5300	53	Glucose 6-phosphate	.0062	1.6085
54	Stearylcarnitine	.0379	1.4525	54	bMCA	.0379	1.2193
55	Imidazolepropionic acid	.0456	1.0449				

*ACNO* asiaticoside–nitric oxide hydrogel, *AC* asiaticoside, *DM* diabetes mellitus, *VIP* Variable importance in projection

### Screening of potential biomarkers and analysis of metabolic pathways

**Figure 6:**
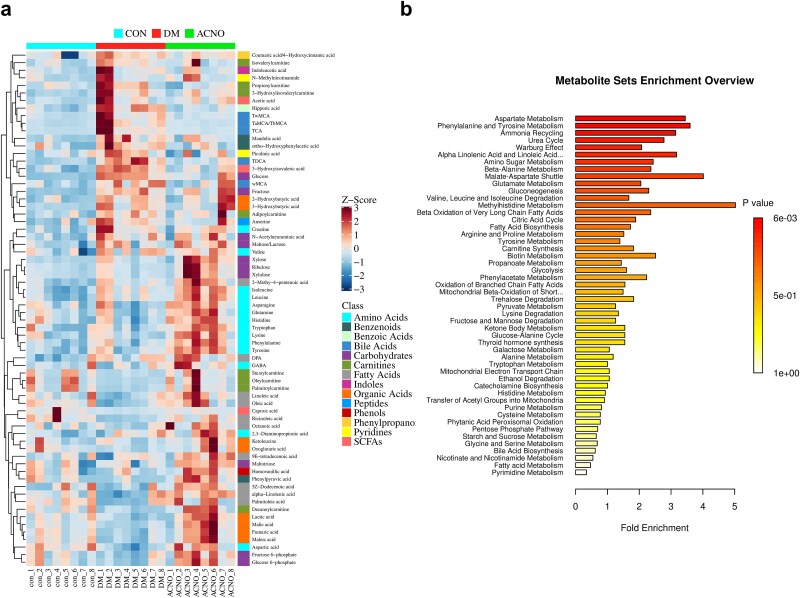
Comparison of differentially expressed metabolite levels among CON group, DM group, and ACNO group. (a) Each row corresponds to data for a specific metabolite, and each column represents a CON, DM, or ACNO group. The color indicates the relative expression levels of the metabolites in this set of samples. (b) Pathway enrichment analysis of differential metabolites. The top 10 metabolic pathways include methyl histidine metabolism, malate–aspartate shuttle, phenylalanine and tyrosine metabolism, aspartate metabolism, alpha-linolenic acid and linoleic acid metabolism, ammonia recycling, urea cycle, amino sugar metabolism, beta-alanine metabolism, and gluconeogenesis. *ACNO* asiaticoside–nitric oxide hydrogel, *AC* asiaticoside, *CON* control, *DM* diabetes mellitus

The potential biomarkers were screened based on the following criteria: VIP > 1 in multidimensional analysis, *P* < .05 in single-dimensional analysis, and |log_2_FC| ≥ 0. The resulting differential metabolites were considered as potential biomarkers and are presented in Table 2. The heat maps in Figure 6a depict the relative strength of biomarkers and the expression levels of metabolites between groups, particularly focusing on the regulatory effect of the ACNO group on DW mice. The findings demonstrate significant changes in the DM group compared to the CON group, with increased levels of metabolites such as 3-hydroxyisovaleric acid, glucose, carnosine, fructose, and pyridine carboxylic acid. Conversely, the levels of specific metabolites, including organic acids, stearyl choline, palmitoyl carnitine, aspartic acid, and certain fatty acids, were reduced. Following drug intervention, ACNO significantly restored the levels of bile acid metabolites, mandelic acid, lactic acid, 3-hydroxyisovaleric acid, selected amino acids, and other metabolites in DM mice (Figure 6a).

The SMPDB database was utilized to perform pathway enrichment analysis on the differential metabolites. In Figure 6b, the color depth represents the *P*-value, with a smaller *P*-value indicating a higher enrichment of differential metabolites in that pathway compared to others. Several metabolic pathways were identified, including aspartic acid metabolism, phenylalanine, and tyrosine metabolism, urea cycle, β-alanine metabolism, and methyl histidine metabolism, among others.

### Network pharmacological analysis

**Figure 7:**
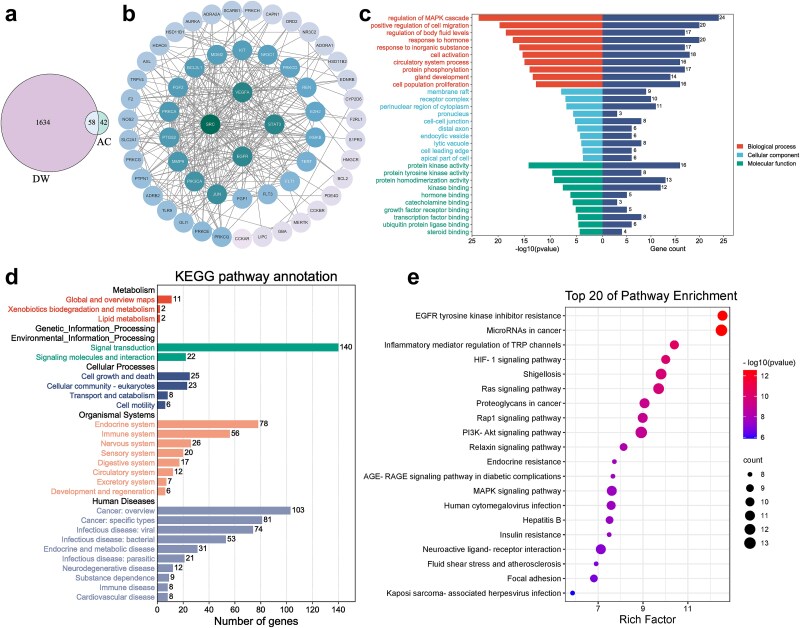
The mechanism of asiaticoside in treating DWs was analyzed by network pharmacology. (a) Venn diagram of potential targets for DWs and asiaticoside. (b) PPI (protein–protein interaction) network diagram. (c) GO enrichment analysis (biological process, cellular component, molecular function). (d) KEGG pathway annotation. (e) Top 20 KEGG pathways of potential targets. *KEGG* Kyoto encyclopedia of genes and genomes, *GO* gene ontology

A total of 1692 targets associated with DWs and 100 targets related to asiaticoside were identified. By taking the intersection of these two sets, 58 common targets were determined as potential targets of asiaticoside in the treatment of DWs. A Venn diagram (Figure 7a) was generated to visualize these potential targets for further analysis. Potential protein–protein–target interaction relationships were constructed using the STRING database. A visual mapping **(**Figure 7b) was created, highlighting the four targets with the highest degree value: SRC, STAT3, EGFR, and VEGFA. It is noteworthy that these four targets are deemed to be pivotal targets of asiaticoside in the management of DWs. The Metascape tool was employed to carry out GO enrichment analysis in order to explicate the biological features of putative targets. The top 10 enriched biological processes included the regulation of MAPK cascade, stimulation of cellular migration, and control of bodily fluid levels, among other significant processes. The top 10 enriched cell components encompassed membrane raft, receptor complex, perinuclear region of cytoplasm, pronucleus, and others. The top 10 enriched molecular functions involved protein kinase activity, protein tyrosine kinase activity, protein homogenization activity, and others (Figure 7c).

To extend the investigation of possible targets, an analysis based on the KEGG was executed to reveal the functional actions of asiaticoside in the management of wounds caused by diabetes. The classification statistics of signaling pathways, as well as the 20 most significant signaling pathways, are exhibited in Figure 7d and e, respectively. The results of KEGG enrichment analysis demonstrate that the key pathways implicated in AC’s anti-DW effect encompass microRNAs in cancer, PI3K-Akt signaling pathway, EGFR tyrosine kinase inhibitor resistance, Rap1 signaling pathway, MAPK signaling pathway, and HIF-1 signaling pathway, among other pathways. According to the pathway classification statistics, asiaticoside exerts its anti-DW effects primarily through the endocrine system, immune system, cell growth and death, signal transduction, and other activities.

### Molecular docking results of asiaticoside with SRC, STAT3, EGFR, and VEGFA

**Figure 8:**
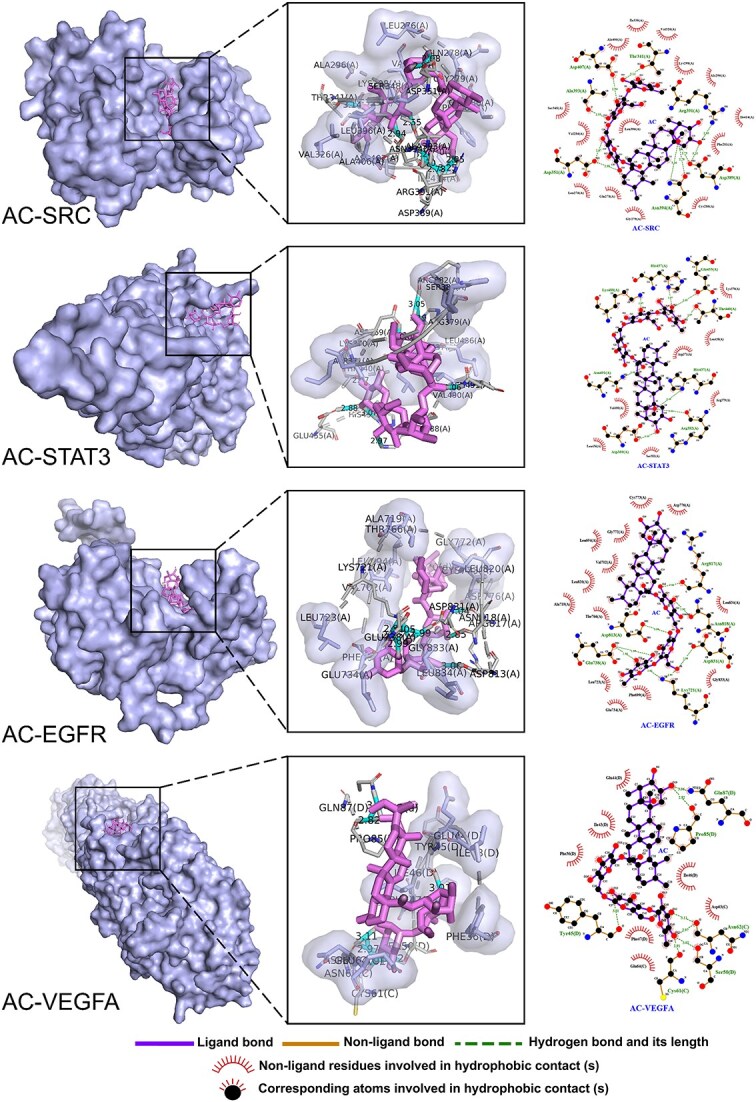
Molecular docking–predicted binding mode. Molecular docking of asiaticoside with SRC (PDB ID: 7NG7), STAT3 (PDB ID: 6NUQ), EGFR (PDB ID: 1 M17), and VEGFA (PDB ID: 4ZFF) proteins. *AC* asiaticoside; SRC, sarcoma, *STAT3* signal transducer and activator of transcription 3, *EGFR* epidermal growth factor receptor, *VEGFA* vascular endothelial growth factor A

Based on the constructed PPI network, four distinct targets were identified for molecular docking. The findings of this docking process, as well as the resultant binding of AC to each of these targets, are compiled and presented in Table 3. Binding energy is a crucial parameter in molecular docking as it helps assess the strength of interaction between two molecules. A lower binding energy indicates a higher affinity between the receptor and ligand. The results of the molecular docking process demonstrate that asiaticoside exhibits a highly favorable binding activity with these key targets. It is especially noteworthy that the binding energy of asiaticoside to EGFR and SRC is −10.1 and −10.3 kcal/mol, respectively. This demonstrates a close and highly active binding potential. The binding modes of asiaticoside to the key targets are depicted in Figure 8. Hydrogen bonding and hydrophobic forces primarily contribute to the interaction between the small molecule (AC) and the protein. AC forms hydrogen bonds with amino acids such as Asp407 (A) and Thr341 (A) on SRC while engaging in hydrophobic interactions with Lys298 (A) and Leu396 (A). Hydrogen bonding occurs between asiaticoside and amino acids such as His457 (A) and Lys488 (A) on STAT3, accompanied by hydrophobic interactions with Val490 (A) and Ser381 (A). AC interacts with amino acids on EGFR through hydrogen bonds with Asp813 (A) and Glu738 (A), as well as hydrophobic interactions with Phe699 (A) and Leu723 (A). Lastly, AC forms hydrogen bonds with amino acids such as Pro85 (D) and Ser50 (D) on VEGFA and engages in hydrophobic interactions with Phe47 (D), and Glu64 (C).

**Table 3:** Molecular docking score (kcal/mol)

**Target name**	**Ligand name**	**Docking score**
SRC	Asiaticoside	−10.3
STAT3		−6.3
EGFR		−10.1
VEGFA		−7.4

*SRC* sarcoma, *STAT*3 signal transducer and activator of transcription 3, *EGFR* epidermal growth factor receptor, *VEGFA* vascular endothelial growth factor A

### ACNO hydrogel restored the expression levels of SRC, STAT3, EGFR, and VEGFA in diabetic wounds to the normal control group

**Figure 9:**
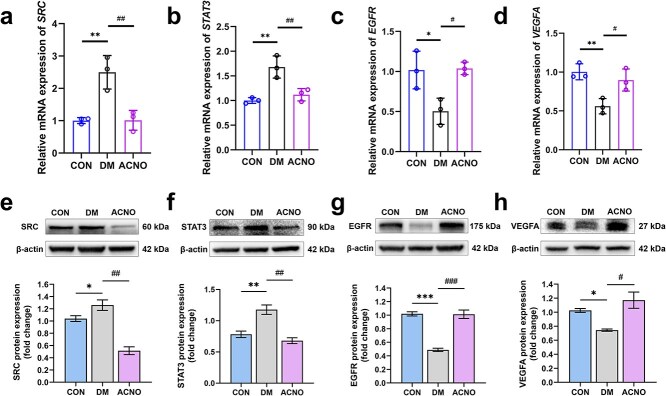
Modulation of SRC/STAT3 signaling pathway by ACNO treatment in DW healing. The mRNA levels of (a) SRC, (b) STAT3, (c) EGFR, and (d) VEGFA were quantified using RT-qPCR, while the corresponding protein levels for (e) SRC, (f) STAT3, (g) EGFR, and (h) VEGFA were determined through western blotting analysis. β-Actin served as an internal control to ensure accurate quantification and comparison across samples. ^*^p < 0.05, ^**^p < 0.01, ^***^p < 0.001 *vs* CON; ^#^*p* < 0.05, ^##^*p* < 0.01, ^###^*p* < 0.001 *vs* DM. Data are presented as mean ± SD, *n* = 3. *SRC* sarcoma, *STAT3* signal transducer and activator of transcription 3, *EGFR* epidermal growth factor receptor, *VEGFA* vascular endothelial growth factor A

To authenticate molecular docking results, we used RT-qPCR to measure the mRNA expression levels of the target protein in three distinct groups: CON, DM, and ACNO. The data presented in Figure 9a–d indicate that the DM group exhibited a marked upregulation in the mRNA expression levels of *SRC* and *STAT3* relative to the CON group. Conversely, *EGFR* and *VEGFA* expression levels were notably diminished in the DM group when juxtaposed with the CON group. Intriguingly, the administration of ACNO hydrogel effectively mitigated these deviations, restoring the expression levels of *SRC*, *STAT3*, *EGFR*, and *VEGFA* in DWs toward those observed in the normal control group. This suggests that ACNO treatment can rectify the altered gene expression associated with DW pathology, aligning with the molecular interactions predicted by docking studies.

We demonstrated that the administration of ACNO hydrogel effectively normalized the expression levels of key proteins involved in the SRC/STAT3 signaling pathway, which were altered in DWs. This finding is significant as it highlights the potential mechanism through which ACNO exerts its therapeutic effects, aligning with our earlier observations of improved wound healing outcomes.

The normalization of SRC, STAT3, EGFR, and VEGFA expression levels suggests that ACNO may modulate these pathways to promote tissue repair and regeneration. This is consistent with previous studies that have identified the SRC/STAT3 pathway as a critical regulator of inflammation and healing processes [32, 33]. By restoring the balance of these signaling molecules, ACNO may enhance cellular responses necessary for effective wound closure and tissue remodeling.

### ACNO improves diabetic wounds by regulating SRC/STAT3 signaling pathway

Protein expression levels of SRC, STAT3, EGFR, and VEGFA were quantified via Western blotting, revealing significant variations across experimental cohorts. Specifically, SRC expression was elevated in the DM group but reduced following ACNO treatment relative to the control (CON) group. A similar pattern was observed for STAT3, with increased expression in the DM group and a decrease post-ACNO treatment. Conversely, EGFR and VEGFA expression levels demonstrated a decline and an upsurge, respectively, in the DM group after ACNO intervention compared to the CON group. These statistically significant alterations elucidate potential molecular mechanisms warranting further exploration (Figure 9e–h).

**Figure 10:**
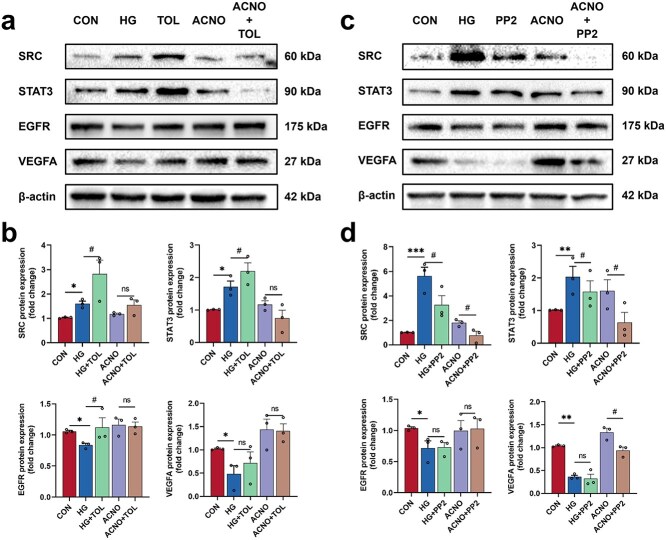
Western blot results of SRC agonist (tolimidone) or SRC inhibitor (PP2). (a) Western blot of SRC, STAT3, EGFR, and VEGFA in HaCaT cells after ACNO and SRC agonist tolimidone treatment. (b) Effect of ACNO and SRC agonist tolimidone treatment on the levels of SRC, STAT3, EGFR, and VEGFA protein expression. (c) Western blot of SRC, STAT3, EGFR, and VEGFA in HaCaT cells after ACNO and SRC inhibitor PP2 treatment. (d) Effect of ACNO and SRC inhibitor PP2 treatment on the levels of SRC, STAT3, EGFR, and VEGFA protein expression. ^*^p < .05, ^**^p < .01, ^***^p < .001 *vs* CON; ^#^*p* < .05, ^##^*p* < .01, ^###^*p* < .001 *vs* HG. Data are presented as mean ± SD, *n* = 3. *SRC* sarcoma, *STAT3* signal transducer and activator of transcription 3, *EGFR* epidermal growth factor receptor, *VEGFA* vascular endothelial growth factor A, *TOL* tolimidone

To investigate the role of SRC activity in the therapy of DWs with ACNO, we designed *in vitro* experiments to analyze the effect of ACNO treatment on the expression of proteins such as SRC, STAT3, and other proteins in HaCaT cells within a hyperglycemic environment, using either an SRC agonist (Tolimidone) or an SRC inhibitor (PP2) (Figure 10).

Our results demonstrated that SRC protein expression was activated in HaCaT cells following treatment with tolimidone, which was accompanied by increased protein expression levels of STAT3 and EGFR, while no changes were observed in the protein expression levels of VEGFA. Compared to the ACNO group, no statistically significant alterations in protein expression levels were noted in the ACNO + TOL group, suggesting that ACNO treatment may have inhibited the activation of Tolimidone, resulting in a marked reduction in SRC protein expression. Consequently, this may have hindered the connection between SRC and downstream signaling molecules.

Similarly, SRC protein expression was inhibited in HaCaT cells after PP2 treatment, coinciding with a decrease in STAT3 expression levels, while there was no effect on the expression levels of EGFR and VEGFA proteins. Treatment of HaCaT cells with ACNO led to a significant reduction in SRC protein expression, and following synergistic effects with PP2, SRC bands were no longer detectable in the ACNO+PP2 group. Furthermore, a comparison of the ACNO group with the ACNO+PP2 group revealed a notable reduction in STAT3 and VEGFA protein expression, likely due to the absence of SRC. In conclusion, these findings indicate that SRC signaling is a prerequisite for ACNO to exert its therapeutic effects in DW healing.

### Comprehensive analysis of metabolomics and network pharmacology

**Figure 11:**
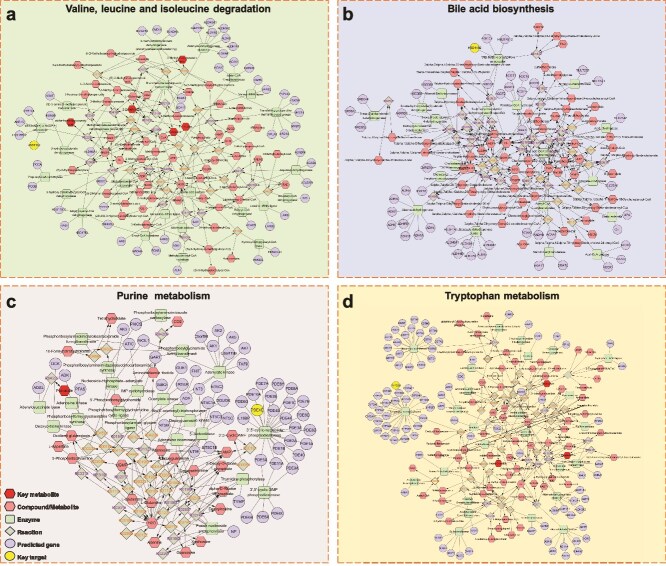
The metabolite-reaction-enzyme-gene network diagram was constructed by integrating the differential metabolites obtained from ACNO hydrogel treatment of DWs in mice and the target of asiaticoside on DWs (predicted by network pharmacology). (a) Valine, leucine, and isoleucine degradation. (b) Bile acid biosynthesis. (c) Purine metabolism. (d) P tryptophan metabolism. Red hexagon, pink hexagon, green rectangle, gray diamond, purple circle, and yellow circle represent key metabolites, compounds/metabolites, enzymes, reactions, predicted genes, and key targets, respectively. *ACNO* asiaticoside–nitric oxide hydrogel, *DW* diabetic wound

The MetScape plug-in was used to correlate the different metabolites found in metabolomics with the drug targets of traditional Chinese medicine components, focusing on key genes. The obtained results are shown in Figure 11. Three targets are screened from the network diagram, namely, HSD11B2, PDE4D, and CYP2D6. Twelve metabolites were identified: isoleucine, oxoglutaric acid, fumaric acid, indoleacetic acid, etc. Among them, four metabolic pathways are involved, namely, valine, leucine, and isoleucine degradation; bile acid biosynthesis; purine metabolism; and tryptophan metabolism (Table 4).

**Table 4:** Information on key targets, metabolites, and pathways

**No.**	**Related pathways**	**Key targets**	**Key metabolites**
1	Valine, leucine, and isoleucine degradation	HSD11B2	Isoleucine Oxoglutaric acid Leucine Ketoleucine Valine
2	Bile acid biosynthesis	HSD11B2	TCA TDCA
3	Purine metabolism	PDE4D	Fumaric acid Aspartic acid Glutamine
4	Tryptophan metabolism	CYP2D6	Indoleacetic acid Oxoglutaric acid Tryptophan

*TCA* taurocholate acid, *TDCA* tauroursodeoxycholic acid

## Discussion

DM represents a complex metabolic anomaly that manifests with heightened levels of blood glucose and impaired wound healing, leading to increased susceptibility to infections in individuals with compromised skin integrity [34]. While the detrimental effects of the impaired inflammatory response on wound healing in DM are widely recognized, our understanding of the immune regulatory mechanisms underlying the impaired healing or nonhealing DWs remains limited. Currently, there is a growing body of research focusing on the utilization of active ingredients derived from Chinese medicine for the treatment of DWs. These active ingredients have shown promising effects in wound healing by influencing fibroblast proliferation and angiogenesis [35]. Furthermore, studies have indicated that Chinese medicine’s active components also affect wound scarring, apoptosis, and inflammatory reactions [36].

In our previous investigations, our research group has established the beneficial role of asiaticoside in enhancing the healing process of DWs using both *in vivo* and *in vitro* experimental models. In the context of wound healing, particularly in DWs, the formation of eschars is a common occurrence. Eschars are dry, dark scabs or crusts that form over a wound as part of the natural healing process. They serve as a protective barrier against infection and further injury, allowing the underlying tissue to regenerate. In our study, the presence of eschars was carefully monitored as an indicator of the wound healing progression. The eschars were observed to form within the expected timeframe and were consistent with the healing stages described in the literature [9, 37]. Regular assessments were conducted to ensure that the eschars did not impede the healing process, and any signs of infection or delayed healing were promptly addressed. The formation and resolution of eschars were documented as part of the overall evaluation of the therapeutic effects of the ACNO hydrogel.

While our study provides preliminary evidence suggesting that ACNO hydrogel may promote hair follicle regeneration, it is important to emphasize that these findings are exploratory and descriptive. The observed increase in hair follicle numbers in the ACNO-treated groups compared to the diabetic control group indicates a potential therapeutic effect. However, the underlying mechanisms remain to be fully elucidated. We acknowledge that further studies, including detailed mechanistic investigations and additional biomarker analyses, are necessary to substantiate these initial observations.

Expanding on the aforementioned discoveries, the current investigation endeavors to clarify the fundamental mechanism of action by means of the amalgamation of metabolomics and network pharmacology. Our objective is to acquire a comprehensive comprehension of the molecular pathways implicated in the therapeutic benefits of asiaticoside on the healing of DWs through the examination of differential metabolites and metabolic pathways.

Lactic acid, identified as one of the potential biomarkers in our study, holds significant importance in the context of DW healing. It serves as an essential energy substrate that fulfills the heightened metabolic demands associated with the wound-healing process [38]. Moreover, the accumulation of lactic acid contributes to a lower pH environment, which creates favorable conditions for cellular proliferation and differentiation within an optimized physiological pH range. Notably, several studies highlighted the role of lactic acid in stimulating collagen synthesis by fibroblasts and promoting angiogenesis in stem cells through redox system modulation [39, 40].

The findings of this study revealed that ACNO significantly restored the levels of lactic acid metabolites in DW mice. Furthermore, the expressions of TCA and TDCA were down-regulated in the CON and ACNO groups, while their expressions were up-regulated in the DM group. These observations suggest that ACNO exerted a significant inhibitory effect on the expression of TCA and TDCA, potentially promoting the healing process. TCA and TDCA involve the metabolic pathway of bile acid biosynthesis. In the “metabolite-reaction-enzyme-gene” network diagram, through the combined analysis of metabolomics and network pharmacology, TCA and TDCA are critical metabolites related to the gene HSD11B2.

Among the top nine endogenous metabolites, glucose was also identified. The expression of glucose metabolites was up-regulated in the DM group, whereas it was down-regulated in the CON and ACNO groups. These findings suggest that the ACNO group exhibited an inhibitory effect on glucose metabolism.

This study identified two core metabolic pathways: methyl histidine metabolism and the malate–aspartic acid shuttle. The malate–aspartic acid shuttle, also known as the malate shuttle, is a biochemical system present in eukaryotic cells that facilitates the transport of electrons released during glycolysis across the semipermeable mitochondrial inner membrane for oxidative phosphorylation [41].

Indeed, the results obtained from molecular docking provide a preliminary understanding of the potential binding interactions between asiaticoside and the target proteins, such as SRC, STAT3, EGFR, and VEGFA. SRC protein is a nonreceptor tyrosine kinase, which can be activated by multiple signal transduction pathways, and the activated SRC kinase can be activated by phosphorylating the tyrosine residue of the corresponding target protein, thus activating the corresponding signal pathways, including MAPK, STAT, PI3K/AKT, and EGFR [42]. STAT3 activation has been linked to endoplasmic reticulum stress in endothelial cells induced by hyperglycemia and may contribute to vascular injury through the upregulation of inflammation and angiogenesis-related genes like VEGF and HIF-1 [43]. EGFR, the specific receptor for EGF, is implicated in the autocrine or paracrine regulation of DW formation and development [44]. VEGFA, a member of the growth factor family, specifically enhances the migration, proliferation, and angiogenesis of vascular endothelial cells [45]. It is crucial to emphasize that these findings are preliminary and require further experimental verification to substantiate the binding efficacy and influence of asiaticoside on these proteins.

RT-qPCR analyses indicated that ACNO could rectify the aberrant gene expression of *SRC*, *STAT3*, *EGFR,* and *VEGFA*. Western blotting results revealed significant differences in the expression of these proteins between the diabetic and control groups. This suggests that modulating the expression of these target proteins is vital for enhancing wound healing in diabetic chronic wounds. Post-ACNO treatment, the expression levels of the four target proteins approached those observed in the CON group, indicating a recovery from the altered protein expression seen in the DM group.

**Figure 12:**
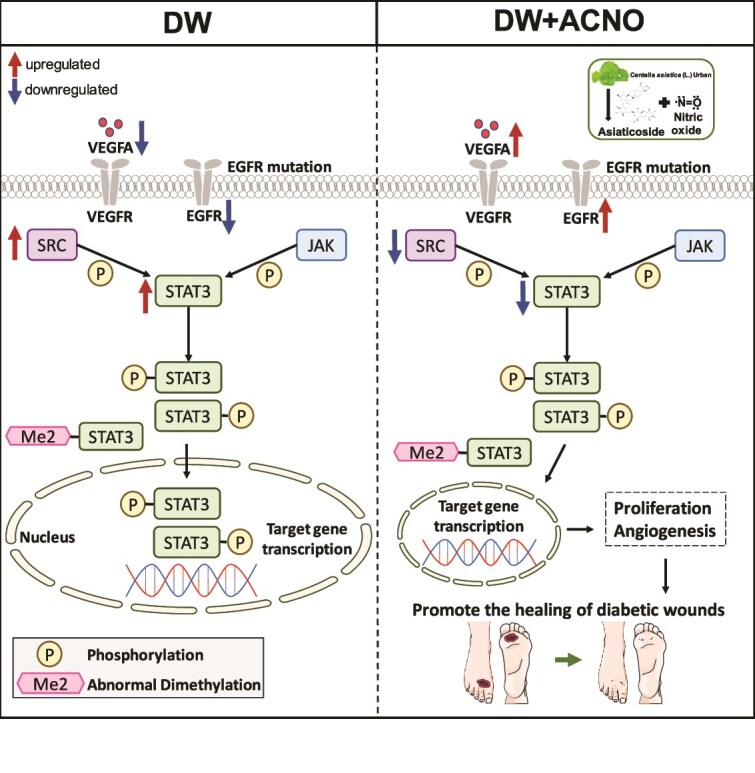
The mechanism diagram of ACNO in treating DWs. *ACNO* asiaticoside–nitric oxide hydrogel, *DW* diabetic wound, *SRC* sarcoma, *STAT3* signal transducer and activator of transcription 3, *EGFR* epidermal growth factor receptor, *VEGFA* vascular endothelial growth factor A

ACNO may promote DW healing by inhibiting the SRC/STAT3 signaling pathway. Figure 12 reveals the mechanism diagram of ACNO in treating DW. At present, the role of the SRC/STAT3 signaling pathway in diabetic complications is not clear. SRC is upstream of STAT3, and the activation of SRC can trigger STAT3 activity and promote the expression of phosphorylated STAT3 [46]. A large number of studies show that inhibiting SRC can inhibit the activation of STAT3 [47]. While the current research offers significant insights into the total protein expression changes induced by ACNO-hydrogel, it is important to note that the detection of SRC/STAT3/EGFR/VEGFA in this study represents a preliminary exploratory experiment. It should be acknowledged that post-translational modifications, such as phosphorylation and methylation [48, 49], are critical for a comprehensive understanding of the signaling pathways involved in DW healing. Incorporating these analyses in future studies will facilitate a more detailed elucidation of the molecular mechanisms through which ACNO hydrogel exerts its therapeutic effects, thereby enhancing both the robustness and applicability of the findings presented in this study.

STAT3 is widely involved in the regulation of cell cycle and apoptosis and affects endothelial activation and vascular inflammation [46, 50]. It is speculated that ACNO can inhibit the expression of proinflammatory factors by inhibiting the SRC/STAT3 signaling pathway, thus promoting the healing of DWs. This study should also detect the effects of proinflammatory factors such as IL-6, IL-1β, and TNF-α. VEGFA/STAT3 signaling pathway plays an anti-inflammatory role by inhibiting the transcription of pro-inflammatory genes and starts an effective repair mechanism [51].

The increased expression of SRC and STAT3 mRNA in the DM group suggests their involvement in initiating angiogenesis during skin injury. ACNO treatment appears to alleviate wounds and reduce inflammation in diabetic mice [52]. Activating JAK/STAT3 signal transduction can regulate the expression of iNOS in HaCaT cells, and in diabetic skin, the JAK/STAT3-mediated upregulation of iNOS can lead to increased NO production [53]. Since NO is known to influence angiogenesis and is beneficial for wound repair [13], the combination of AC and NO in this study could represent a significant advancement in the treatment of DWs.

Network pharmacology offers a method to predict drug targets at an overall level by integrating vast amounts of data [54]. However, it is subject to false positives, necessitating validation through additional methods such as metabolomics and transcriptomics to enhance accuracy [55]. Limitations of network pharmacology and related databases include the need for more accurate and comprehensive target information, low correlation between drug components and their targets across databases, and a need for improved website maintenance and stability.

We successfully combined the target genes predicted by network pharmacology with the pathways and differential metabolites predicted by metabolomics. Among them, isoleucine involved in the metabolic pathway of “degradation of valine, leucine, and isoleucine” can induce the expression of antibacterial peptides, which opens up a new way for the development of anti-infective drugs. Studies have shown that isoleucine can be combined with nicotinyl, valine, and histidine to form a new peptide, which shows the effect of improving wound healing under hyperglycemia [56]. It mainly promotes wound healing by up-regulating the expression of antioxidant genes in HaCaT cells. In addition, fumaric acid, which is involved in the purine metabolism pathway, can be used to cross-link carboxymethyl cellulose (CMC)–chitosan (CSN) hydrogel and treat various skin diseases, including psoriasis and skin wounds [57]. At present, it has been studied that fumaric acid is mixed into Ag/agar-agar hybrid hydrogel, and it is found that fumaric acid can promote antioxidation and angiogenesis pathways [58]. Hydrogels containing fumaric acid also exhibit potential antibacterial effects against common pathogens and can encourage granulation tissue formation and angiogenesis, thereby aiding wound healing.

In addition, glutamine, which is also related to the purine metabolic pathway, is the most abundant amino acid in human plasma. It is used as an energy source for cell proliferation, including fibroblasts, macrophages, and epithelial cells. Some studies show that glutamine can increase the concentration of arginine and citrulline (the precursor of arginine), which will promote wound healing [59]. The wound healing pathway involves the arginase pathway, which produces polyamines, as well as ornithine and proline. The former is related to cell proliferation, while the latter is related to collagen synthesis.

In the binding analysis, tryptophan metabolism is also closely related to asiaticoside in treating DW mice. Studies have shown that exogenous tryptophan can promote the migration and AKT phosphorylation of dermal fibroblasts and promote the skin wound healing of chronic stress mice by inhibiting TNF-α and IDO activation [60]. This multifaceted approach, which includes the use of asiaticoside, NO, and amino acid modulation, presents a comprehensive strategy for improving DW healing.

In future studies, it is recommended to conduct receptor purification and *in vitro* affinity analysis to determine the binding affinity between asiaticoside and the target proteins. Furthermore, performing point mutation experiments can aid in identifying the specific amino acid residues that are crucial for the binding interaction. This will contribute to a more comprehensive understanding of the binding mechanism and validate the active sites predicted based on the docking model.

## Conclusions

The present study demonstrates the efficacy of ACNO hydrogel in promoting DW healing by reversing metabolic dysregulation and modulating key molecular pathways. Using UPLC-MS/MS and molecular docking approaches, researchers identified key metabolites (including mandelic acid, lactic acid, and 3-hydroxyisovaleric acid) and critical signaling pathways involved in ACNO’s wound-healing mechanism. The findings revealed that ACNO effectively ameliorated metabolic perturbations in DW tissues by modulating the methyl histidine metabolism and malate–aspartate shuttle pathways. Notably, ACNO promoted angiogenesis and cell proliferation through regulation of the SRC/STAT3 signaling pathway, as evidenced by immunofluorescence staining and molecular analyses. The study demonstrated ACNO’s ability to normalize the expression of crucial proteins (SRC, STAT3, EGFR, and VEGFA) and identified potential therapeutic targets (HSD11B2, PDE4D, and CYP2D6), establishing a comprehensive mechanistic framework for ACNO’s efficacy in DW healing and providing a scientific foundation for its potential pre-clinical application.

## Data Availability

The data that support the findings of this study are available from the corresponding author upon reasonable request.
